# Development of Toddlers’ Smartphone Flow State Scale: Parent Report Form

**DOI:** 10.3390/ijerph182211833

**Published:** 2021-11-11

**Authors:** Mikyong Byun, GyeongAe Seomun

**Affiliations:** 1 Department of Nursing Science, College of Medicine, Catholic Kwandong University, 24 Beomil-ro 579beon-gil, Gangneung-si 25601, Korea; mulanbb@korea.ac.kr; 2 College of Nursing, Korea University, Anam-dong, Seongbuk-gu, Seoul 02841, Korea; 3 BK21FOUR R&E Center for Learning Health Systems, Korea University, Seoul 02841, Korea

**Keywords:** toddler, smartphone, flow, scale

## Abstract

Toddlers come into contact with smartphones by the time they are 10 months old, and smartphones eventually become a part of the daily lives of toddlers because they are used as parenting tools and are also attractive toys. Routine exposure to these electronic devices may lead to excessive immersion, which can cause smartphone dependence when toddlers grow up. Based on Csikszentmihalyi’s concept of flow, we aimed to develop a new scale to measure the flow phenomenon in toddlers who are exposed to smartphones. We attempted to identify the constituent factors of a toddler’s flow in smartphones through a literature review, observations, and interviews. Initially, 32 questions were screened from the field verification stage and interviews; the final 20 questions were selected by combining technical statistics, exploratory factor analysis, and expert feasibility. We also found five eligible constituent factors, namely, a playfulness-oriented experience, reaction limited by concentration, and intentional pursuit to achieve the goal, assimilate into the virtual world, and acquire desire-fulfilling skills. We then performed a confirmatory factor analysis on our parent-reported toddlers’ smartphone flow state scale. To secure the criterion-related validity, the correlation between our scale and the preexisting smartphone dependence measurement tool for toddlers was evaluated. Cronbach’s α value of the toddlers’ smartphone flow state scale was 0.95 (each factor was verified as 0.79–0.92 and the explanatory power was 72.21%). The test–retest reliability was found to be stable with the intraclass correlation (ICC) coefficient value of 0.78 (*p* < 0.001). Our research findings suggest that this novel smartphone flow state scale for toddlers could be a valid and reliable tool for measuring how toddlers feel the flow phenomenon while using smartphones. Furthermore, our results could contribute to the development and evaluation of the interventions that prevent side effects from smartphone overflow in toddlers.

## 1. Introduction

Smartphones are portable electric devices that integrate telephone and computing functions into one unit, which have become increasingly popular and even indispensable. Smartphones have become more commonly used as parenting tools due to parents’ convenience and toddlers’ preferences. As a result, we can easily observe toddlers immersing themselves in these attractive “toys” in several locations, such as restaurants, strollers, and cars [1]. A recent study found an incidence of first smartphone use of 45.1% at the age of one (12–24 months) and 20.2% at the age of two (24–36 months); smartphones become a part of daily life by the time toddlers are 36 months old [2]. The daily smartphone usage time of Korean 24-month-old toddlers has been shown to be 57 min in those receiving daycare services and 84 min toddlers not receiving these services. Considering smartphone-based learning such as audiovisual education, overall phone exposure to toddlers is more than two hours per day [3].

Smartphone dependence refers to a condition in which a person overuses a smartphone and displays withdrawal and intolerance of phone use, thereby suffering disturbances in their daily life [4]. Toddlers can also feel uneasy when they cannot use smartphones for any reason, and sometimes cannot engage in other activities unless smartphones are within reach. However, some researchers suggest that it may not be appropriate to define this phenomenon as “dependence” in toddlers, because sustained withdrawal and intolerance are rarely seen in toddlers according to their developmental stage [5,6]. Research works on toddlers also recommend to refrain from using negative terms for various reasons such as stigma effects [7,8]; hence, we adopted the concept of “smartphone flow” instead of “smartphone dependence” in this study.

Flow is a state of immersion in an activity. This concept has been continuously revised since it was introduced by Csikszentmihalyi in 1975. Based on this theory, Jackson et al. developed the first flow state scale, which was validated by Mundell [9,10]. This measurement tool is now recognized as the first tool with reliability and validity in the field of psychology and has been used in various situations, including immersion research or related topics [11,12,13,14]. Moneta and Csikszentmihalyi stated that flow measurement should be independently applied depending on the area and activity [15].

Based on the theory of flow, we first attempted to identify the constituent factors of toddlers’ smartphone flow through a literature review and interviews. We then developed a new scale to measure the flow phenomenon in toddlers exposed to smartphones. Finally, we aimed to verify the validity and reliability of our scale in the real world to provide a theoretical basis for the development and evaluation of intervention programs that can prevent possible side effects from smartphone overflow in toddlers.

## 2. Materials and Methods

**Figure 1:**
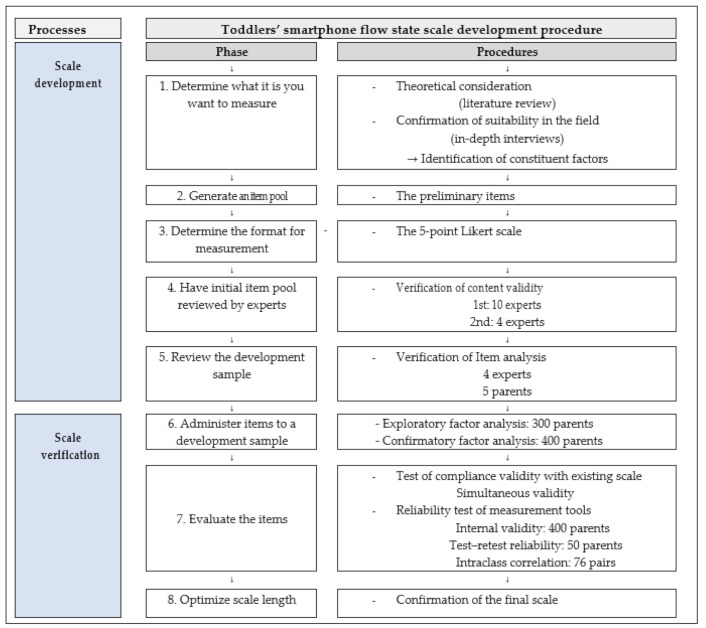
Research procedure. The Toddlers’ Smartphone Flow State Scale (TS-FSS) was developed and validated according to DeVellis’ eight stages of scale development.2.2. Generate an Item Pool.

The development and validation of the Toddlers’ Smartphone Flow State Scale (TS-FSS) were conducted as per the eight stages of scale development and verification [16] (Figure 1).

### 2.1. Determine What Is Measured

To develop a measurement tool, a conceptual definition of the flow was established. To this end, we sought to clarify this concept by reviewing the relevant literature and collecting theoretical and empirical evidence. We systemically reviewed literature works published from 1975 to 2019 using three international databases (PubMed, PsycINFO, and CINAHL) and two Korean databases (KISS and RISS). The main search terms were “flow,” “indulgence,” “immersion,” “engagement,” “addiction,” and “dependence.” Research works that met the following criteria were eligible: (a) Papers must relate to the search items above; (b) papers must be published from 1975 to 2019; (c) papers must relate to the components of flow, which can be utilized in our flow state scale. 

In the preliminary stage, we performed direct home interviews with eight parents of children aged 12–36 months. Examples of the interview questions are as follows: “Have you ever observed that your child finds pleasure and fun while using a smartphone?” and “Have you ever found your child paying attention to a smartphone?” Based on the literature review and the interview results, the constituent factors were confirmed. 

Three international databases (PubMed, PsycINFO, and CINAHL) and two Korean databases (KISS and RISS) were also used for the literature review. The search terms included “toddlers,” “patients,” “population,” “smartphone,” “flow,” “immersion,” “addiction,” “exposure,” and “intervention.” Articles that met the following criteria were eligible: (a) Papers must deal with toddlers; (b) papers must relate to the search items above; (c) papers must have been published from 1996 to 2019. Based on a literature review and the contents from field suitability, preliminary questions on toddlers’ smartphone flow were prepared. Edward’s informal criteria for attitude statements were followed [17]. For the sake of readability, complex meanings were not included, and one question did not encompass multiple situations [16].

### 2.2. Determine the Format for Measurement

The experience sampling method (ESM), interviews, the observation method, and a questionnaire are usually used to measure flow. Since the first introduction of the flow state scale by Jackson et al., questionnaires are commonly used nowadays [9,10]. As for the scoring method of the measurement, the greater the number of steps, the higher the reliability. The reliability increased when steps 5, 6, and 7 were applied but not step 4; there was no difference among steps 5, 6, and 7 [18,19,20,21]. In this study, we used a five-point Likert scale to measure the flow state scale. 

### 2.3. Review by Experts

Two rounds of expert validation were conducted. The first validation was performed by 10 experts: Three mental health nursing professors, two professors of child nursing, two professors whose main research area is scale development, one pediatrician, one pediatric psychiatrist, and one child counseling center director. The second expert validation was determined by one professor whose main research area is scale development, one professor with early childhood smartphone intervention experience, one mental health nursing professor, and one director of a child counseling center. 

Both the item-level content validity index (I-CVI) and scale-level content validity index (S-CVI) scores were evaluated [22,23].

### 2.4. Review of the Development Samples

When preparing questions for measurement scales, the level of language was tailored according to the survey subject [24]. A questionnaire review was conducted to check whether there were any items that could not convey meaning, were difficult to understand, or were difficult to answer. Four experts who participated in the second validation and five parents who participated in the interview conducted this review process.

### 2.5. Administer Items

A recruitment notice was posted on the online communities for parents. Parents voluntarily participated in this study through interviews or online surveys (using the SurveyMonkey program). A total of 700 parents were enrolled; 300 parents belonged to EFA and the other 400 belonged to CFA.

Construct validity was evaluated by item analysis, exploratory factor analysis (EFA), and confirmatory factor analysis (CFA). Item analyses of the preliminary items were conducted to select items with a correlation coefficient ≥0.4 [25]. For the EFA, Kaiser–Meyer–Olkin (KMO) and Bartlett’s spherical tests were used to determine whether the preliminary items were suitable for factor analysis [26]. A factor analysis was conducted with principal component analysis and promax rotation [27,28]. As a criterion for extracting the appropriate factors from the principal component analysis, we measured the eigenvalue of the item and a factor loading ≥0.40 was selected [29,30]. A CFA was performed through a structural equation model [31]. Maximum likelihood estimation was used [32], and goodness-of-fit was determined by considering the absolute and incremental fit indices [33,34].

### 2.6. Evaluate the Items

Evaluation of criterion validity. Concurrent validity was measured using the “Smartphone dependence measurement tool for 36 months to 9 years old” by the National Information Society Agency Statistics and Research Report Korea (2016). It consists of three components: (a) Salience (three items), (b) self-control failure (three items), and (c) serious consequences (three items). The higher the score, the higher the degree of dependence on smartphones, and the overall reliability of the tool was found to be Cronbach’s α 0.86 [4]. Discriminant validity was reviewed by the Fornell–Larcker criterion [35].

Evaluation of the reliability. To verify the internal consistency and reliability, Cronbach’s α was calculated [36]. Furthermore, test–retest reliability was confirmed by deriving intraclass correlations (ICCs)−a method of evaluating the agreement between repeated measurements [37,38]. Additionally, inter-rater reliability was also measured [39,40].

### 2.7. Optimize Scale Length

We optimized scale items through validity and reliability testing. The final TS-FSS was established based on its reliability, validity, and expert opinions [16].

## 3. Results

### 3.1. Identifying the Constituent Factors

Our search initially returned 1354 papers (PubMed, 1167; PsycINFO, 12; CINAHL, 16; KISS, 37; and RISS, 122). After a full-text review by the researchers, we included 22 articles matching the aim of our study. Since the initial definition of flow by Csikszentmihalyi, various definitions of flow have been suggested and revised by many scholars. This concept has been continuously revised and supplemented since Csikszentmihalyi first described six factors of experiencing flow in 1975 [40]. Recent studies have proposed nine factors of flow [15,41,42]: (a) Coherent, noncontradictory task demand and clear; (b) demand for action and clear feedback; (c) challenge–skill balance; (d) control of actions and environment; (e) merging action awareness; (f) centering of attention on a limited stimulus; (g) loss of ego; (h) altered sense of time; and (i) autotelic experience [15,40,41,42,43]. In this study, a measuring scale of a toddler’s smartphone flow was developed using the flow components of Csikszentmihalyi, being the most comprehensive and widely used concept among researchers. Interviews with eight parents of toddlers were conducted, and suitability was verified by confirming whether toddlers experience smartphone flow in real life as shown in the literature.

### 3.2. The Preliminary Items

Based on the nine components of flow theory, our empirical contents through observation and interviews were prepared. Considering that the number of items required for final measurement is 1.5–2 times [16], a total of 32 preliminary items were registered.

### 3.3. Five-Point Likert Scale

The questions used a five-point Likert scale, a widely used scale incorporated to measure the flow state scale [9,11,12,44,45].

### 3.4. Verification of Content Validity

The first validation panel consisted of 10 experts, and items with a validity index of less than 0.78 were generally removed. Some items with a validity index of 0.7 could possibly be utilized after modification following expert opinion. The second validation panel was composed of four experts; as a result of the second expert validity verification, the item-level content validity index was measured to be 0.80 or higher. 

### 3.5. Verification of Item Analysis

We received feedback from five parents and four experts by e-mail. Queries were modified to make them easier to understand.

### 3.6. Exploratory and Confirmatory Factor Analyses 

A total of 700 parents were enrolled. Three hundred parents belonged to EFA; the other 400 belonged to CFA. The general characteristics are listed below (Table 1). 

Item analysis. The mean, standard deviation, skewness, and kurtosis of each TS-FSS item were analyzed. Skewness and kurtosis values are reported within ±2 for all 28 items [46] (Table 2).

The overall mean value of the items ranged from 2.38 to 3.53, with a standard deviation of 0.908–0.109. After checking the internal reliability when an item was dropped, GO3 and EX3 were removed. Both LO1 and LO2 showed a low expert validity score of 0.8 and were deemed as similar to AT (centering attention on a limited stimulus) according to the expert opinion. As a result, the LO1 and LO2 questions were removed.

Exploratory factor analysis. The KMO value of TS-FSS was 0.952 (*χ*^2^ = 5946.529, df = 378, *p* < 0.001), showing a suitability above 0.9, and Bartlett’s sphericity test showed statistical significance, confirming that it was suitable for factor analysis. In the same manner of deep correlation among factors in Csikszentmihalyi’s flow theory, our correlation results among components were also high in this study. Thus, the promax method was applied for factor rotation [28].

Initiated by Guttman and propagated by Kaiser [27], there is a criterion that the eigenvalue should be greater than or equal to 1.0; however, in cases of small samples, the number of factors tends to be overestimated, so that the value is not absolute and sometimes meaningful eigenvalues can be less than 1.00 [40,47,48].

In our analysis, the eigenvalue was 1.088 with four factors; however, clear goals and task demands, clear feedback (which are factors that promote commitment), and self-purposed experiences (which is a factor resulting from commitment) were reduced to one factor, which is not appropriate in theory. On the contrary, the eigenvalue with five factors was 0.895. Although this value did not satisfy the criterion, we determined that five factors (i.e., a playfulness-oriented experience, reaction limited by concentration, intentional pursuit to achieve the goal, assimilation into the virtual world, and acquisition of desire-fulfilling skills) were more appropriate according to the theory. The explanatory variance, factor loading, and cumulative variance for the final 20 items are as shown below (Table 3).

To determine whether the five factors (which originated from factor analysis) measure each component, the correlations between factors were evaluated. In this study, the correlation coefficient between factors was found to be less than 0.85.

**Figure 2:**
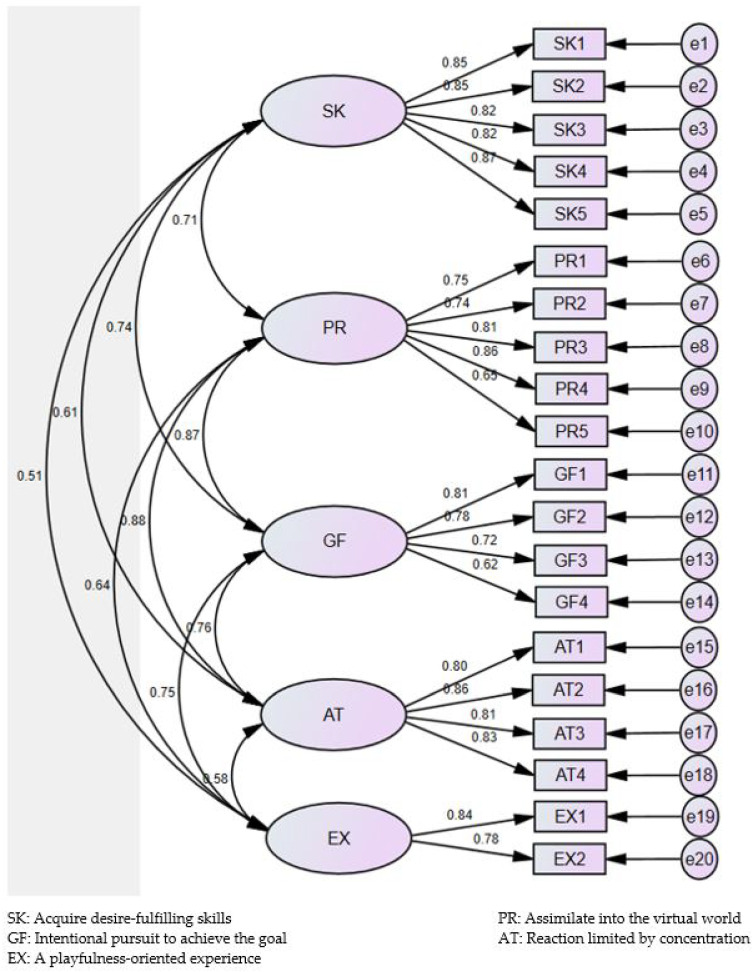
Confirmatory factor analysis model of TS-FSS (*n* = 400).

Confirmatory factor analysis. Data from 400 parents raising 12–36-month-old toddlers were available in the analysis without any missing information. To carry out the confirmatory factor analysis, the model was estimated using the maximum likelihood method. This model was established based on the five factors derived from the exploratory factor analysis. As a result, χ^2^ was found to be 633.595 (df = 160, *p* < 0.001), and when other absolute fit indices were checked, the root mean square residual (RMR) value was 0.033. In addition, the goodness-of-fit index (GFI), incremental fit index (IFI), Tucker–Lewis’s index (TLI), and comparative fit index (CFI) values exceeded the value of 0.8. Therefore, our parent-reported toddlers’ smartphone flow state scale seemed to achieve model suitability (Table 4). The TS-FSS measurement model is shown in Figure 2.

When the average variance extracted (AVE) is 0.50 or more and the construct reliability (CR) is 0.70 or more, it could be considered to have adequate concentration validity in confirmatory factor analysis [46,49]. Concentrated validity was verified as the variance extraction index of this research tool met the condition of 0.50 or more. The concept reliability was found to be 0.7 or more (Table 5).

### 3.7. Test of Validity and Reliability

Discriminant validity. This was reviewed by the Fornell–Larcker criterion, and was considered to be secured when all AVE square roots presented on the diagonal were greater than the correlation values between latent variables below the diagonal [35]. It can be said that the discriminant validity is secured between the two factors. In this study, discriminant validity was secured as shown below (Table 6).

Concurrent validity. To confirm the simultaneous validity of TS-FSS, the correlation with the smartphone dependence measurement tool developed by the Korea Internet and Security Agency [50] was evaluated. This preceding smartphone dependence measurement tool was made for children, adolescents, and adults. We could identify and analyze a total of 72 eligible cohorts from that report, which matched our targeted population of 36-month-old toddlers. The correlation coefficients between our scale and the previous tool are shown below and indicate a statistically significant correlation (Table 7).

Internal consistency reliability. Cronbach’s α value of the TS-FSS was 0.95, and the value of each factor ranged from 0.79 to 0.92 (Table 8).

Test–retest reliability. The TS-FSS was repeatedly administered to 50 participants to examine the test–retest reliability, and the same number of participants were finally included in the analysis. The ICC coefficient was found to be 0.992 (*p* < 0.001) [37,38].

Inter-rater reliability. The purpose of checking the inter-rater reliability is to estimate the consistency between the scores measured by multiple evaluators. To demonstrate that the evaluations were objective, the degree of agreement between the raters was calculated and reported. There was no absolute standard for recognizing the reliability of the evaluation data for the degree of agreement between evaluators, but a correlation coefficient of 0.6 or higher was observed when the evaluation result was given as a score [18,19,51,52].

### 3.8. Confirmation of the Final Scale

After going through eight steps of Devellis, we confirmed five key factors and 20 eligible questions in our TS-FSS. 

## 4. Discussion

A toddler usually encounters a smartphone as a toy by the time they are 10 months old, and this attractive electric device then becomes a part of their daily routine. In this study, the concept of toddlers’ smartphone flow (rather than the concept of smartphone dependence) was defined as a state in which the smartphone itself provides purpose, satisfaction, and complete immersion when toddlers use the device. Inspired by Csikszentmihalyi’s flow theory, we designed a new tool to measure the flow phenomenon that toddlers experience while using smartphones. Based on the immersive factors by Csikszentmihalyi [42], survey items were selected through a theoretical literature review, interviews, and observations. We identified five key factors, and the final 20 questions were eligible through expert validations, content analysis, technical statistics, and exploratory/confirmatory factor analyses.

The five constituent factors include a playfulness-oriented experience, reaction limited by concentration, intentional pursuit to achieve the goal, assimilation into the virtual world, and acquisition of desire-fulfilling skills. First, “A playfulness-oriented experience” is to feel pleasure and entertainment through the flow of smartphones and to feel satisfaction in the activity itself while continuing to flow themselves when using smartphones. This is the driving force that maintains the flow, and toddlers want to continue to use smartphones to achieve playfulness through smartphones. Second, ”Reaction limited by concentration” means that the toddlers limit or narrow their range of perception only to the stimulation of the smartphone, so that they do not respond to other stimuli. While toddlers experience smartphone flow, their attention and concentration increase; however, if they are frequently exposed to smartphone flow, toddlers play through the stimulating perspective of smartphones, making it difficult to stimulate their curiosity for real objects. Frequent smartphone flow in toddlers can make them lose interest in other play formats and reduce creative thinking skills, as they reduce imaginary training opportunities through actual play. Third, “Intentional pursuit to achieve the goal” means that toddlers crave a smartphone and maintain smartphone immersion to obtain playfulness. Toddlers’ ability develops through specific objects and actual feedback—when no immediate reaction is made to the action, the flow is broken. The immediate response of the smartphone to toddlers’ behavior is an important factor in maintaining their smartphone flow state. Fourth, “Assimilate into the virtual world” means that toddlers become assimilated to the virtual reality provided by smartphones. It makes them unable to recognize their own real existence and lose sense of time. When toddlers see their favorite characters, they experience flow to the point that they cannot attend to anything other than what they are seeing. Fifth, “Acquire desire-fulfilling skills” refers to a toddler’s smartphone use skill that is acquired to obtain playfulness. Toddlers’ motor development is initially dependent on parents, but gradually, they learn to freely control their bodies and maintain balance. The developmental characteristics of toddlers who want to act independently and the time when they first start using smartphones are intertwined. When toddlers learn to control smartphones themselves by operating them with two hands, their degree of smartphone flow increases. We could assume that frequent and repeated experiences of smartphone flow might lead to overdependence later on. Likewise, we observed that the higher the TS-FSS score, the higher the incidence of a toddler’s smartphone flow in our study.

As toddlers are unable to participate in the flow survey on their own, we adopted a novel parent-reported toddlers’ smartphone flow measurement instead. The main subjects of this research were parents or caregivers of 12–36-month-old toddlers. This parent-reporting measurement tool is an observer scale by which parents directly observe and report their children’s behavior. As mentioned above, our TS-FSS includes five main factors with 20 specific survey items. The reliability of Cronbach’s α value was 0.95 and the explanatory power was 72.21%. We must keep in mind that a toddler’s smartphone use is usually determined by parental permission; thus, parents (measurer) are important variables in the design of preventive interventions against excessive smartphone immersion [53,54,55]. To supplement the limitations of the observer measurement tool, which is not a self-written measurement tool, the inter-rater reliability verification process was conducted in this study. We confirmed that our TS-FSS is a measurement scale with secured inter-rater reliability.

The pre-existing smartphone measurement tool was made for all ages and primarily based on a literature review and expert opinions [50]. We developed a new tool for a specific population (toddlers and their parents) on the theoretical basis of Csikszentmihalyi’s flow. As is well known, this theory-based measurement scale has the advantage of securing content validity and reliability. The concept of flow has been continuously updated since its first introduction; Abuhamdeh et al. suggested that the method of flow measurement might be modified in various situations, areas, and subjects. In other words, measurement scales need to be developed accordingly. In terms of media environments, including virtual reality, Hoffman et al. discussed the importance of continuing efforts to measure the flow phenomenon [14,56]. For example, Apple started the smartphone era with the iPhone, which combined a touch screen and a user-friendly interface in 2007. As smartphones have gained popularity, a new paradigm on media related to this electronic device has begun. Moreover, another new form of media will be prevalent, but Csikszentmihalyi’s concept of flow would remain useful and applicable to any media, situation, area, or subject in the future.

## 5. Conclusions

Our research findings suggest that our parent-reported smartphone flow state scale for toddlers could be a valid and reliable tool for measuring how toddlers feel the flow phenomenon while using smartphones. We also observed a possible link leading to smartphone dependence if the flow phenomenon in toddlers persists. Furthermore, our results could contribute to the development and evaluation of interventions that prevent side effects resulting from smartphone overflow. Our results necessitate further validations by well-designed studies with a larger cohort in the future. 

## Figures and Tables

**Table 1:** Respondents’ general characteristics (*n* = 700).

Variable	Category	EFA *		CFA **	
		*n*	(%)	*n*	(%)
Respondent’s gender	Male (father) Female (mother)	37 263	12.3 87.7	180 220	45 55
Child’s age	13–20 months 21–28 months 29–36 months	84 90 126	28.0 30.0 42.0	111 110 179	27.7 27.5 44.8
Child’s gender	Male (boy) Female (girl)	150 150	50 50	205 195	51.3 48.7
Child’s birth order	1st 2nd 3rd 4th or more	196 96 7 1	65.3 32.0 2.3 0.3	297 89 12 2	74.3 22.2 3.0 0.5
Time spent in one go	10 min or less 11–30 min 31–60 min 1 hour or more	102 109 67 22	34.0 36.3 22.3 7.3	134 121 93 52	33.5 30.2 23.3 13
Average times of use (per day)	30 min or less 31–60 min 61–120 min 121–180 min 3 h or more	120 94 63 19 4	40.0 31.3 21.0 6.3 1.3	139 98 106 36 21	34.7 24.5 26.5 9.0 5.3
	Total (*n*)	300		400	

* EFA, exploratory factor analysis; ** CFA, confirmatory factor analysis.

**Table 2:** Descriptive statistics and internal consistency in the exploratory factor analysis of TS-FSS (*n* = 300).

Question		Min	Max	Mean	SD	Skewness	Kurtosis	Item–Total Correlation Coefficient	Reliability If an Iitem Is Dropped	Item Internal Consistency
Factor	Code									
GO	GO1	1	5	3.53	0.908	−0.828	0.778	0.687	0.638	0.780
	GO2	1	5	3.36	0.983	−0.382	−0.517	0.626	0.694	
	GO3 **	1	5	2.91	1.090	−0.141	−0.781	0.556	0.782	
FE	FE1	1	5	3.65	0.941	−0.819	0.706	0.572	0.753	0.810
	FE2	1	5	3.73	0.865	−0.721	0.659	0.572	0.787	
CH	CH1	1	5	2.91	1.055	0.078	−0.469	0.746	0.748	0.844
	CH2	1	5	2.91	1.082	−0.050	−0.772	0.694	0.799	
	CH3	1	5	2.87	1.017	−0.205	−0.542	0.692	0.800	
AT	AT1	1	5	3.15	1.083	−0.229	−0.738	0.705	0.832	0.865
	AT2	1	5	2.99	1.043	−0.158	−0.712	0.736	0.819	
	AT3	1	5	2.58	0.997	0.206	−0.639	0.725	0.824	
	AT4	1	5	2.58	1.017	0.319	−0.536	0.694	0.836	
ME	ME1	1	5	2.88	1.018	−0.256	−0.712	0.725	0.787	0.850
	ME2	1	5	2.49	1.126	0.201	−1.015	0.757	0.754	
	ME3	1	5	2.94	1.063	−0.125	−0.732	0.680	0.827	
CO	CO1	1	5	2.72	1.064	0.126	−0.732	0.751	0.794	0.862
	CO2	1	5	2.65	1.064	0.132	−0.802	0.758	0.787	
	CO3	1	5	2.38	1.006	0.511	−0.238	0.706	0.835	
LO	LO1 *	1	5	2.40	0.985	0.219	−0.873	0.589	0.794	0.818
	LO2 *	1	5	2.69	1.097	0.050	−0.904	0.636	0.772	
	LO3	1	5	2.91	1.131	−0.311	−0.941	0.692	0.746	
	LO4	1	5	2.87	1.185	−0.062	−0.902	0.647	0.769	
TI	TI1	1	5	2.85	1.025	−0.296	−0.789	0.701	0.650	0.794
	TI2	1	5	3.08	1.027	−0.472	−0.365	0.646	0.711	
	TI3	1	5	2.26	1.034	0.540	−0.486	0.568	0.792	
EX	EX1	1	5	3.40	0.988	−0.745	0.353	0.741	0.633	0.803
	EX2	1	5	3.69	0.915	−0.866	0.946	0.651	0.734	
	EX3 **	1	5	2.80	1.097	−0.040	−0.835	0.573	0.823	
GO: Coherent, noncontradictory task demand and clear CH: Challenge–skill balance ME: Merging action awareness LO: Loss of ego EX: Autotelic experience							FE: Demand for action and clear feedback AT: Centering of attention on a limited stimulus CO: Control of actions and environment TI: Altered sense of time			
LO1 * and LO2 *: Deletion by expert validity							GO3 ** and EX3 **: Deletion by reliability if an item is dropped			

**Table 3:** Loading of TS-FSS items in the exploratory factor analysis (*n* = 300).

Factor	No. of Item	Factor Loading Value				
		1	2	3	4	5
A playfulness-oriented experience	CO1 ME2 CO2 CH3 CH2	0.885 0.843 0.795 0.778 0.730	0.506 0.038 0.520 0.500 0.498	0.531 0.584 0.461 0.557 0.555	0.530 0.465 0.497 0.595 0.573	0.400 0.386 0.389 0.514 0.444
Reaction limited by concentration	LO4 LO3 TI1 TI3 TI2	0.432 0.498 0.539 0.485 0.590	0.818 0.790 0.763 0.690 0.688	0.487 0.472 0.663 0.583 0.670	0.334 0.448 0.417 0.289 0.534	0.473 0.538 0.543 0.344 0.665
Intentional pursuit to achieve the goal	GO2 GO1 FE1 FE2	0.536 0.539 0.538 0.495	0.509 0.580 0.555 0.451	0.816 0.804 0.767 0.762	0.438 0.370 0.451 0.450	0.393 0.436 0.646 0.683
Assimilate into the virtual world	AT4 AT3 AT2 AT1	0.500 0.494 0.459 0.402	0.369 0.403 0.313 0.287	0.406 0.438 0.308 0.393	0.769 0.745 0.735 0.631	0.411 0.484 0.513 0.514
Acquire desire-fulfilling skills	EX1 EX2	0.485 0.368	0.560 0.461	0.531 0.415	0.539 0.515	0.821 0.786
	Eigenvalue (%)	6.919	6.155	6,684	5.657	5.740
	Variance (%)	44.690	6.572	5.850	3.751	2.348
	Cumulative variance (%)	46.489	55.032	62.600	60.039	72.208
GO: Coherent, noncontradictory task demand and clear CH: Challenge–skill balance ME: Merging action awareness LO: Loss of ego EX: Autotelic experience		FE: Demand for action and clear feedback AT: Centering of attention on a limited stimulus CO: Control of actions and environment TI: Altered sense of time				

**Table 4:** Fit indices of TS-FSS in the confirmatory factor analysis (*n* = 400).

	*χ* ^2^	df	RMR	GFI	NFI	IFI	TLI	CFI	RMSEA
TS-FSS	633.595	160	0.033	0.888	0.904	0.917	0.902	0.917	0.086
									(0.079–0.093)
RMR: Root mean square residual TLI: Tucker–Lewis’s index			GFI: Goodness-of-fit index CFI: Comparative fit index				IFI: Incremental fit index RMSEA: Root mean square error of approximation		
TS-FSS: Toddlers’ Smartphone Flow State Scale									

**Table 5:** Concentrated validity for TS-FSS (*n* = 400).

Factor	Item			AVE	CR
Acquire desire-fulfilling skills	SK1	My child is good at handling smartphones the way he wants.		0.677	0.770
	SK2	My child can find what he wants on a smartphone.			
	SK3	My child has the skills he has learned to use a smartphone.			
	SK4	My child quickly adapts to the new features of the smartphone.			
	SK5	My child uses a smartphone as naturally as an adult.			
Assimilate into the virtual world	PR1	My child imitates or identifies smartphone characters.		0.575	0.740
	PR2	My child uses words on a smartphone without meaning.			
	PR3	My child seems to feel his time differently than usual when using a smartphone.			
	PR4	My child uses a smartphone without knowing the passing of time.			
	PR5	My child was running out of sleep time because he/she was using a smartphone.			
Intentional pursuit to achieve the goal	GF1	My child seems to have a clear reason to look at a smartphone.		0.603	0.705
	GF2	My child has something he wants to do through a smartphone.			
	GF3	My child checks the response of a smartphone while touching the desired point on the screen.			
	GF4	My child immediately reacts to the touch screen that changes screen as soon as he/she touches it.			
Reaction limited by concentration	AT1	My child does not respond when his/her name is called while using a smartphone		0.638	0.718
	AT2	My child does not feel it when he/she is approached while using a smartphone.			
	AT3	My child is enthusiastic about using a smartphone and forgets about other activities.			
	AT4	My child only sees their smartphone the whole time they are using it.			
A playfulness-oriented experience	EX1	My child enjoys using the smartphone itself.		0.580	0.762
	EX2	My child seems to be having fun while using a smartphone.			
SK: Acquire desire-fulfilling skills GF: Intentional pursuit to achieve the goal EX: A playfulness-oriented experience			PR: Assimilate into the virtual world AT: Reaction limited by concentration		

**Table 6:** Discriminant validity for TS-FSS (*n* = 400).

Factor	A Playfulness-Oriented Experience	Reaction Limited by Concentration	Intentional Pursuit to Achieve the Goal	Assimilate into the Virtual World	Acquire Desire-Fulfilling Skills
A playfulness-oriented experience	0.670				
Reaction limited by concentration	0.371	0.868			
Intentional pursuit to achieve the goal	0.416	0.498	0.663		
Assimilate into the virtual world	0.360	0.650	0.539	0.803	
Acquire desire-fulfilling skills	0.340	0.499	0.512	0.571	0.938

Square root value of the variance extracted index.

**Table 7:** Concurrent validity for TS-FSS (*n* = 72).

Concurrent Vailidity		Toddlers’ Smartphone Flow State Scale						Infant Smartphone Dependence Measurement			
		A	B	C	D	E	TS-FSS	a	b	c	IS-DM
Toddlers’ Smartphone Flow State Scale	A	1									
	B	0.689	1								
	C	0.612	0.624	1							
	D	0.383	0.661	0.485	1						
	E	0.392	0.436	0.599	0.295	1					
	TS-FSS	0.795	0.884	0.831	0.746	0.656	1				
Infant Smartphone Dependence Measurement	a	0.607	0.794	0.660	0.742	0.468	0.845	1			
	b	0.517	0.741	0.586	0.632	0.409	0.744	0.703	1		
	c	0.544	0.781	0.483	0.676	0.382	0.746	0.717	0.693	1	
	IS-DM	0.622	0.862	0.643	0.764	0.468	0.869	0.904	0.885	0.899	1
A: Acquire desire-fulfilling skills B: Reaction limited by concentration C: Intentional pursuit to achieve the goal D: Assimilate into the virtual world E: A playfulness-oriented experience							a: Salience b: Self-control failure c: Serious consequences				

**Table 8:** Internal consistency reliability for TS-FSS.

factor		No. of Items	Cronbach’s α
1	A playfulness-oriented experience	4	0.818
2	Reaction limited by concentration	5	0.876
3	Intentional pursuit to achieve the goal	5	0.923
4	Assimilate into the virtual world	4	0.894
5	Acquire desire-fulfilling skills	2	0.791
Toddlers’ Smartphone Flow State Scale		20	0.950

## Data Availability

The data presented in this study are available from the authors upon reasonable request.
